# A Population of Kisspeptin/Neurokinin B Neurons in the Arcuate Nucleus May Be the Central Target of the Male Effect Phenomenon in Goats 

**DOI:** 10.1371/journal.pone.0081017

**Published:** 2013-11-18

**Authors:** Kohei Sakamoto, Yoshihiro Wakabayashi, Takashi Yamamura, Tomomi Tanaka, Yukari Takeuchi, Yuji Mori, Hiroaki Okamura

**Affiliations:** 1 Laboratory of Veterinary Ethology, The University of Tokyo, Tokyo, Japan; 2 Laboratory of Neurobiology, National Institute of Agrobiological Sciences, Ibaraki, Japan; 3 Laboratory of Veterinary Reproduction, Tokyo University of Agriculture and Technology, Tokyo, Japan; University of Rouen, France

## Abstract

Exposure of females to a male pheromone accelerates pulsatile gonadotropin-releasing hormone (GnRH) secretion in goats. Recent evidence has suggested that neurons in the arcuate nucleus (ARC) containing kisspeptin and neurokinin B (NKB) play a pivotal role in the control of GnRH secretion. Therefore, we hypothesized that these neurons may be the central target of the male pheromone. To test this hypothesis, we examined whether NKB signaling is involved in the pheromone action, and whether ARC kisspeptin/NKB neurons receive input from the medial nucleus of the amygdala (MeA)—the nucleus suggested to relay pheromone signals. Ovariectomized goats were implanted with a recording electrode aimed at a population of ARC kisspeptin/NKB neurons, and GnRH pulse generator activity, represented by characteristic increases in multiple-unit activity (MUA) volleys, was measured. Pheromone exposure induced an MUA volley and luteinizing hormone (LH) pulse in control animals, whereas the MUA and LH responses to the pheromone were completely suppressed by the treatment with an NKB receptor antagonist. These results indicate that NKB signaling is a prerequisite for pheromone action. In ovariectomized goats, an anterograde tracer was injected into the MeA, and possible connections between the MeA and ARC kisspeptin/NKB neurons were examined. Histochemical observations demonstrated that a subset of ARC kisspeptin/NKB neurons receive efferent projections from the MeA. These results suggest that the male pheromone signal is conveyed via the MeA to ARC kisspeptin neurons, wherein the signal stimulates GnRH pulse generator activity through an NKB signaling-mediated mechanism in goats.

## Introduction

 In sheep and goats, the exposure of seasonally anestrous females to sexually mature males accelerates reproductive activity and subsequently induces out-of-season ovulation [1-5], a phenomenon called the “male effect.” The male–female interaction consists of several factors, including olfactory, visual, auditory, and tactile/behavioral cues [4,5]. Because exposing females solely to ram fleece or buck hair can induce ovulation, a pheromone released by the male possibly plays a major role in the male effect [5-9] The initial endocrine event following the reception of the male pheromone in the female is an increase in the frequency of episodic luteinizing hormone (LH) secretion. Therefore, the central target of the pheromone signal is believed to be the hypothalamic gonadotropin-releasing hormone (GnRH) pulse generator [2,4,10,11] that governs pulsatile GnRH secretion and thereby pulsatile LH secretion [12]. 

 By recording multiple-unit activity (MUA) in the mediobasal hypothalamus, GnRH pulse generator neural activity has been successfully represented as periodic bursts of MUA (termed “MUA volleys”) in conscious animals such as monkeys [13,14], rats [15,16], and goats [17,18]. Hamada et al. demonstrated that exposure to the male pheromone immediately induced an MUA volley with an accompanying LH pulse in female Shiba goats (*Capra hircus*), confirming for the first time that the pheromone signal indeed activates the GnRH pulse generator [19]. Since this initial report, MUA volley induction has been used as a reliable index to assess pheromone activity in this species [10,20-22]. Shiba goats are nonseasonal breeders under natural daylight [23]. Although the male effect was initially identified as the phenomenon in anestrous females of seasonal breeders [1-5], our previous studies have shown that the induction of the MUA volley by the male pheromone can be observed independent of the season in ovariectomized (OVX) Shiba goats [10,19-22]. In this experimental representation of the male effect, timing of pheromone exposure is critical [10,19]. In this regard, a relatively constant interval of spontaneous MUA volleys in OVX goats [17,18,24] provides practical merit because timing of the next MUA volley can be anticipated, and thus timed pheromone exposure can be done between two successive MUA volleys as described below. The studies in goats unambiguously indicate that the male pheromone activates the GnRH pulse generator. However, no study has identified a specific neuronal population within the mediobasal hypothalamus responsible for MUA volley generation. Thus, the neural identity for the central target of the male pheromone remains unknown.

 Human genetic studies have revealed that kisspeptin and neurokinin B (NKB) are involved in the central control mechanism of GnRH secretion [25-27]. In concert, it has been demonstrated that administration of kisspeptin or NKB (or their analogues) increases LH secretion [28-30], whereas the administration of their antagonists suppresses LH secretion [29,31-33]. A population of neurons in the arcuate nucleus (ARC) co-expresses these 2 neuropeptides in a variety of mammals, including mice [34], rats [35], sheep [36], goats [24], and monkeys [29]. Recent emerging evidence suggests that the population of kisspeptin/NKB neurons in the ARC is a likely candidate for the intrinsic source of the GnRH pulse generator [24,34,37-39]. This hypothesis is supported by findings that the active electrode that detected the MUA volley was indeed located in close proximity to kisspeptin/NKB neurons in the caudal portion of the ARC in both male [40] and female [24] goats. Therefore, it is reasonable to hypothesize that the neural signal of the male pheromone is conveyed to, and processed in, ARC kisspeptin/NKB neurons to facilitate GnRH pulse generator activity.

 To address this hypothesis, the present study aimed to clarify 2 issues. First, because NKB signaling has been suggested to play a pivotal role in GnRH-pulse-generating mechanisms in kisspeptin/NKB neurons [24,38], we investigated whether NKB signaling participates in the pheromone action cascade in OVX Shiba goats. Goats implanted with an MUA recording electrode were treated with the NKB receptor antagonist SB222200 [29], and the effects of the male pheromone on the MUA volley and LH pulse were examined. Second, we investigated neural pathways involved in pheromone signal transduction to ARC kisspeptin/NKB neurons using the tract-tracing method. In general, it has been thought that pheromone signals detected by chemosensory neurons are conveyed to the medial nucleus of the amygdala (MeA). The MeA, in turn, transmits the signals directly or via the bed nucleus of the stria terminalis (BNST) to the hypothalamic nuclei, which controls behavioral and neuroendocrine outputs [41-43]. Furthermore, it has been shown that electrical stimulation or lesion of the MeA [44] modulates LH secretion in rats. Therefore, we injected an anterograde tracer, biotin dextran amine (BDA), to the MeA of OVX goats and examined using BDA histochemical analyses whether the MeA send efferent fibers to the ARC. We also evaluated whether ARC kisspeptin/NKB neurons receive projections from the MeA by using dual-labeling histochemical analyses for BDA and kisspeptin. Because pulsatile GnRH/LH release is indispensable for reproductive success [12], elucidation of neural pathways that facilitate GnRH pulse generator activity, such as the pheromone signal transduction pathway, might contribute to better understanding of the central control mechanism of reproduction in mammals.

## Materials and Methods

### Animals

 Adult female Shiba goats aged 5-7 years were used in this study. They were maintained with a standard pellet diet and dry hay, and had free access to water and supplemental minerals. The Committee on the Care and Use of Experimental Animals at the National Institute of Agrobiological Sciences approved all experimental procedures (#H18-002-1). All efforts were made to minimize animal suffering.

### Effects of the NKB receptor (NK3R) antagonist on pheromone action

 Five goats, ovariectomized at least 6 months prior to the experiment, were implanted with an array of bilateral recording electrodes consisting of six Teflon-insulated platinum-iridium wires (75 µm in diameter) [17,24,40]. The electrode array was implanted targeting the cluster of kisspeptin neurons that are concentrated in the posterior region of the ARC as described previously [24,40]. After recovery, the goats were kept in a condition-controlled room (12L/12D, 23°C, and 50% relative humidity) and loosely held in an individual stanchion throughout the experimental period. MUA was monitored in the conscious goats. Signals were passed through a buffer amplifier integrated circuit directly plugged into an electrode assembly. After further amplification and amplitude discrimination, MUA signals were stored as counts per 20 s on a computer, and the MUA profile was visualized on a display in real time. A characteristic increase in the MUA (MUA volley) was considered the electrophysiological manifestation of the neural element that generates pulsatile GnRH secretion. The occurrence of MUA volleys at relatively constant intervals was confirmed in each goat before an experiment.

 Hair collected from the head region of an adult male goat was used as the pheromone source [10,19,22]. A small hair sample (approximately 1.0 g) was put between 2 plastic cups, in one of which the bottom of the inner cup was replaced with a mesh [10]. The opening of the outer cup was sealed with Parafilm. Upon exposure, the Parafilm was removed, and the goat’s muzzle was inserted into the cup for 10 sec. The cup was then re-sealed and removed from the experimental room immediately.

To examine the possible involvement of NKB signaling in pheromone action, an NK3R antagonist, SB222200 (Sigma-Aldrich, St. Louis, MO, USA), was used. Five mg of the drug was dissolved in 1 mL of 72% dimethyl sulfoxide on the day of the experiment. The dose was determined on the basis of a previous study in monkeys [29], as well as from our pilot studies in goats.

 The 5 goats received the vehicle and SB222200 once, separated by 1 week. On each experimental day, the goats were fitted with a jugular catheter for drug delivery and blood sampling. At first, MUA was monitored for 2 h without blood sampling (control period). The mean intervolley interval during the control period was calculated and used to anticipate the occurrence of the next expected MUA volley in each goat. After 1 regularly occurring MUA volley, the goat was exposed to the pheromone for 10 sec at a time 2/3 of the mean intervolley interval from the preceding MUA volley. SB222200 or vehicle was intravenously injected after the preceding MUA volley. Because neither the half-life of SB222200 in the body nor the time it takes for the drug to reach the putative action site was known, injections were done twice (at the first and fourth blood sampling points) after the preceding MUA volley to ensure that the effective dose of the drug would remain in circulation at the time of pheromone exposure. Blood samples were collected every 6 min for 2 h beginning at the end of the control period, and MUA was monitored throughout the experiment.

### LH assays

 Blood samples were centrifuged at 3000 rpm for 15 min, and plasma was stored at -30°C until used. LH concentrations in 100 µL of plasma were determined in duplicate using a double-antibody radioimmunoassay as described previously [45]. Intra- and inter-assay coefficients were 8.9% and 17.0%, respectively.

 We termed an increase in LH secretion that follows an MUA volley as an LH pulse in this study. Because relatively short inter-pulse intervals in OVX animals gave insufficient time points between the peak and nadir values of LH concentrations, the computer-aid analysis of the LH pulsatility was not performed. 

### Data analyses

 The intervolley interval was the time interval between the start of 2 successive volleys. The start of a volley was determined by an abrupt increase in MUA, which was followed by a sustained increase in MUA for approximately 2 min. The mean intervolley interval during the control period as well as the interval between MUA volleys occurring immediately before and after pheromone exposure was obtained for each goat on each experimental day (vehicle and SB222200 injection). Data are presented as the mean ± SEM in the 5 goats. To analyze effects of the pheromone and SB22220, the 2 parameters were compared using a paired *t* test.

### Anterograde tracer injection into the MeA

 Six goats, ovariectomized at least 1 month prior to the experiment, were used. Tracer injection was done according to a previously described method [46], except that the survival period was increased from 7 to 14 days, taking into account the distance between injection sites and the ARC. Briefly, under halothane anesthesia, a 23-gauge stainless steel guide cannula was placed unilaterally, 4 mm dorsal to the MeA, by referring to specific brain structures such as the lateral ventricle and optic chiasm on radio-ventriculographs. A 30-gauge injector, 4 mm longer than the guide cannula, was then inserted and 10% BDA (MW = 10,000; Life Technologies, Carlsbad, CA, USA) in 10 mM phosphate buffer (PB) was injected at a rate of 10 nL/min for 10 min by using a microinjection pump (model ESP-32; Eicom, Kyoto, Japan). 

 After 14 days, the goats were sacrificed using an overdose of sodium pentobarbital and the heads were perfused bilaterally with 4 L of 10 mM PB (pH 7.4), containing 0.9% sodium chloride, 3000 U heparin/L, and 0.7% sodium nitrate, followed by perfusion of 0.1 M PB containing 4% paraformaldehyde. The brain was dissected rostrally at the organum vasculosum laminae terminalis, caudally at the anterior edge of the mammillary body, and dorsally at the middle of the lateral ventricles. The brain block was immersed in the same fixative overnight at 4°C, followed by immersion in 20% sucrose in 0.1 M PB until the block sank. Frontal sections (50 µm) were cut serially on a freezing microtome, and the sections were maintained in the cryoprotectant solution [47] at -20°C.

### Histochemical analyses

 Single-labeling histochemical analyses for BDA and dual-labeling histochemical analyses for BDA and kisspeptin were performed according to methods described previously [46]. To examine the injection site and distribution of BDA-containing fibers, BDA was detected using the avidin-biotin complex (Vector laboratories, Burlingame, CA, USA) and nickel-intensified 3,3’-diaminobenzidine as the chromogen. BDA-labeled sections were briefly counterstained with cresyl violet (Sigma-Aldrich) or Nuclear Fast Red (Vector Laboratories). The nuclei boundaries were determined on the basis of the appearance of counter-stained cells and the goat brain atlas [48].

 To examine possible projections from the MeA to the ARC kisspeptin neurons, fluorescence immunohistochemical analyses for kisspeptin using the anti-kisspeptin monoclonal antibody (Takeda, no. 245) and Alexa 555-conjugated anti-mouse IgG (Life Technologies) were performed, followed by BDA fluorescence histochemical analyses using the avidin-biotin complex, the tyramide amplification biotin kit (TSA Biotin; PerkinElmer Inc., Waltham, MA, USA), and streptavidin-Alexa 488 (Life Technologies). Specificity of the anti-kisspeptin antibody in goat tissues has been confirmed elsewhere [40]. 

 Sections were observed under a microscope (ECLIPSE E800M; Nikon, Tokyo, Japan) equipped with a charge-coupled device camera (AxioCam HRc; Carl Zeiss, Jena, Germany). In 1 BDA single-labeled section, images in the same field were taken at 6 different focal planes and merged with the aid of computer software (DynamicEye Real; Mitani Corp., Tokyo, Japan) for better presentation of fine structures of BDA-positive products. In dual-labeling histochemical analyses, the 2 fluorescent images were merged using a computer software (AxioVision; Carl Zeiss). Some sections were further analyzed using confocal microscopy (LSM780 or LSM700; Carl Zeiss) with sequential imaging of the 2 channels. Photomicrographs were taken at the same focal plane (1-µm thick), and cells and fibers were considered dual-labeled for BDA/kisspeptin when positive signals overlapped in the same focal plane.

## Results

**Figure 1:**
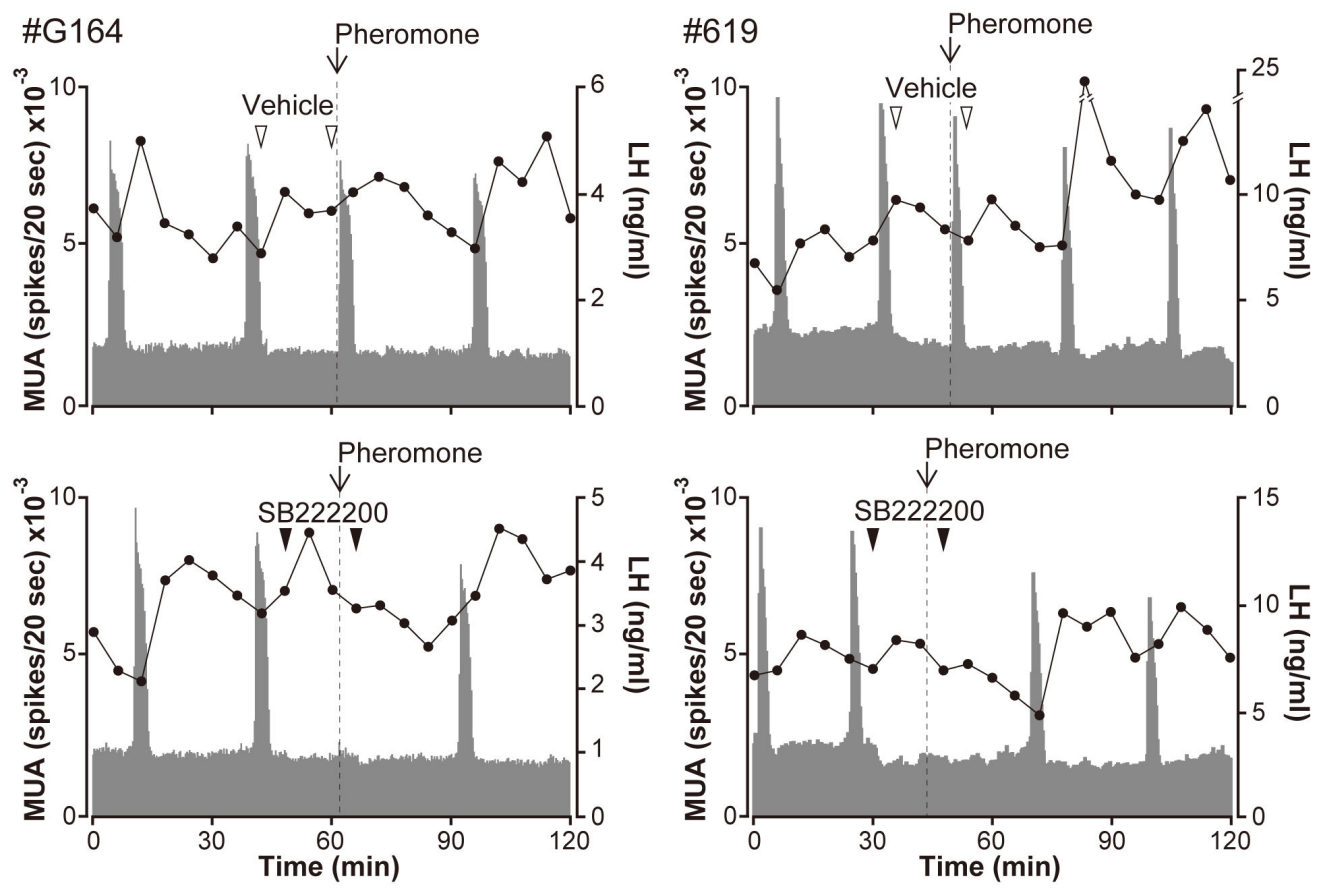
Multiple-unit activity (MUA) profiles and plasma luteinizing hormone concentrations in two representative ovariectomized goats. Goats were exposed to the male pheromone in the absence (upper panels) or presence (lower panels) of the NK3R antagonist SB222200. The timing of pheromone exposure is indicated by an arrow and dotted line. Vehicle (open arrowheads) or SB222200 (closed arrowheads) were injected intravenously twice (at the first and fourth blood sampling points) after the preceding MUA volley.

 To examine the possible involvement of NKB signaling in pheromone action, goats were exposed to the pheromone in the absence or presence of the NK3R antagonist treatment. Two representative examples of MUA profiles and plasma LH concentrations are shown in Figure 1. As expected, pheromone exposure induced an MUA volley accompanied by an LH pulse within 30 s in the 5 vehicle-treated goats, resulting in a significant reduction in the intervolley interval after pheromone exposure than in the control period (21.7 ± 5.2 min vs. 31.0 ± 6.7 min, n=5, p < 0.001). After the pheromone-induced MUA volley, MUA volleys occurred at regular intervals, similar to what was observed in the control period (Figure S1). In contrast, pheromone exposure failed to induce an MUA volley and an LH pulse in the goats treated with SB222200. Compared with the control period, the intervolley interval after pheromone exposure was significantly larger (48.0 ± 6.2 min vs. 29.3 ± 5.0 min, n=5, p < 0.001). There were no apparent changes in the basal MUA profiles after injection of SB222200. 

**Figure 2:**
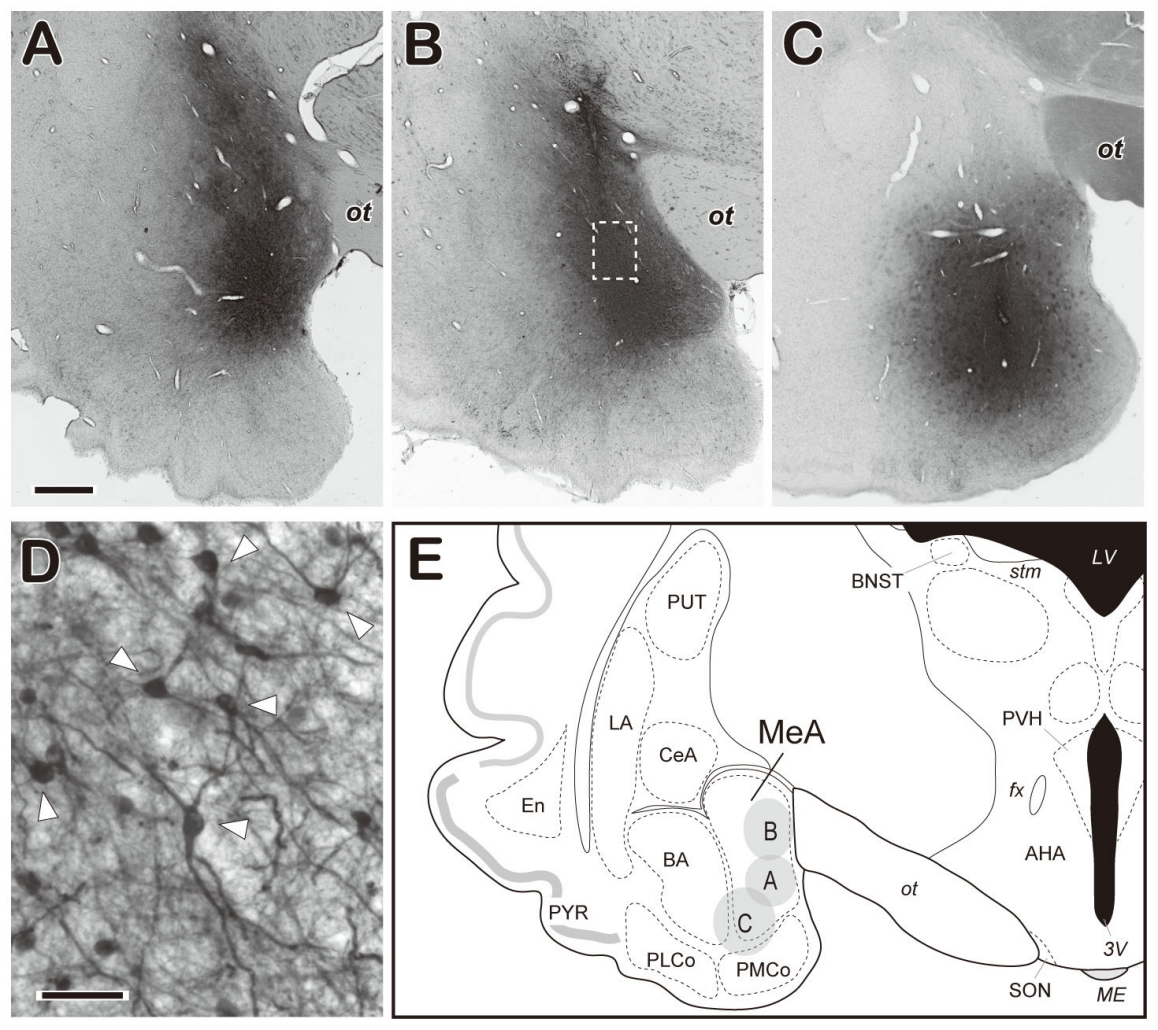
Biotin dextran amine (BDA) injection into the medial nucleus of the amygdala (MeA). Photomicrographs of sections stained for BDA show the injection site in the MeA in 3 ovariectomized goats (A–C). The injection site is characterized by densely packed BDA-positive signals. D, High magnification of the squared area in B. Open arrowheads indicate cells that took up BDA. E, The goat brain atlas, including the amygdaloid complex and the injection site. Shaded areas (A–C) correspond to respective photomicrographs (A–C). Amygdaloid complex: BA, basal nucleus; CeA, central nucleus; MeA, medial nucleus; LA, lateral nucleus; PLCo, posterolateral cortical nucleus; PMCo, posteromedial cortical nucleus; AHA, anterior hypothalamic area. Other structures: BNST, bed nucleus of the stria terminalis; En, endopiriform cortex; PVH, paraventricular nucleus of the hypothalamus; PYR, piriform cortex; PUT, putamen; SON, supraoptic nucleus; *ac*, anterior commissure; *fx*, fornix; *LV*, lateral ventricle; *ME*, median eminence; *ot*, optic tract; *stm*, stria medullaris of the thalamus; *3V*, third ventricle. Scale bar: A = 1 mm for A-C; D = 50 µm.

Figure 2 shows photomicrographs of the injection site in 3 OVX goats, and a goat brain atlas showing the MeA with schematic drawings of the injection site. Densely packed BDA-positive products characterized the injection site, which was elliptical or round in shape, with an approximate diameter of 1.5–3 mm. Of the 6 goats used in the tract-tracing study, 2 had the injection site confined to the MeA (Figure 2A, 2B, and 2E), and 1 had the injection site located in the ventrolateral region of the MeA, with some extension into the adjacent basal amygdala and posterior medial cortical amygdala (Figure 2C and 2E). A higher magnification shows a number of cells that had taken up BDA at the injection site (Figure 2D). In the remaining 3 goats, the injection site did not contain the MeA.

**Figure 3:**
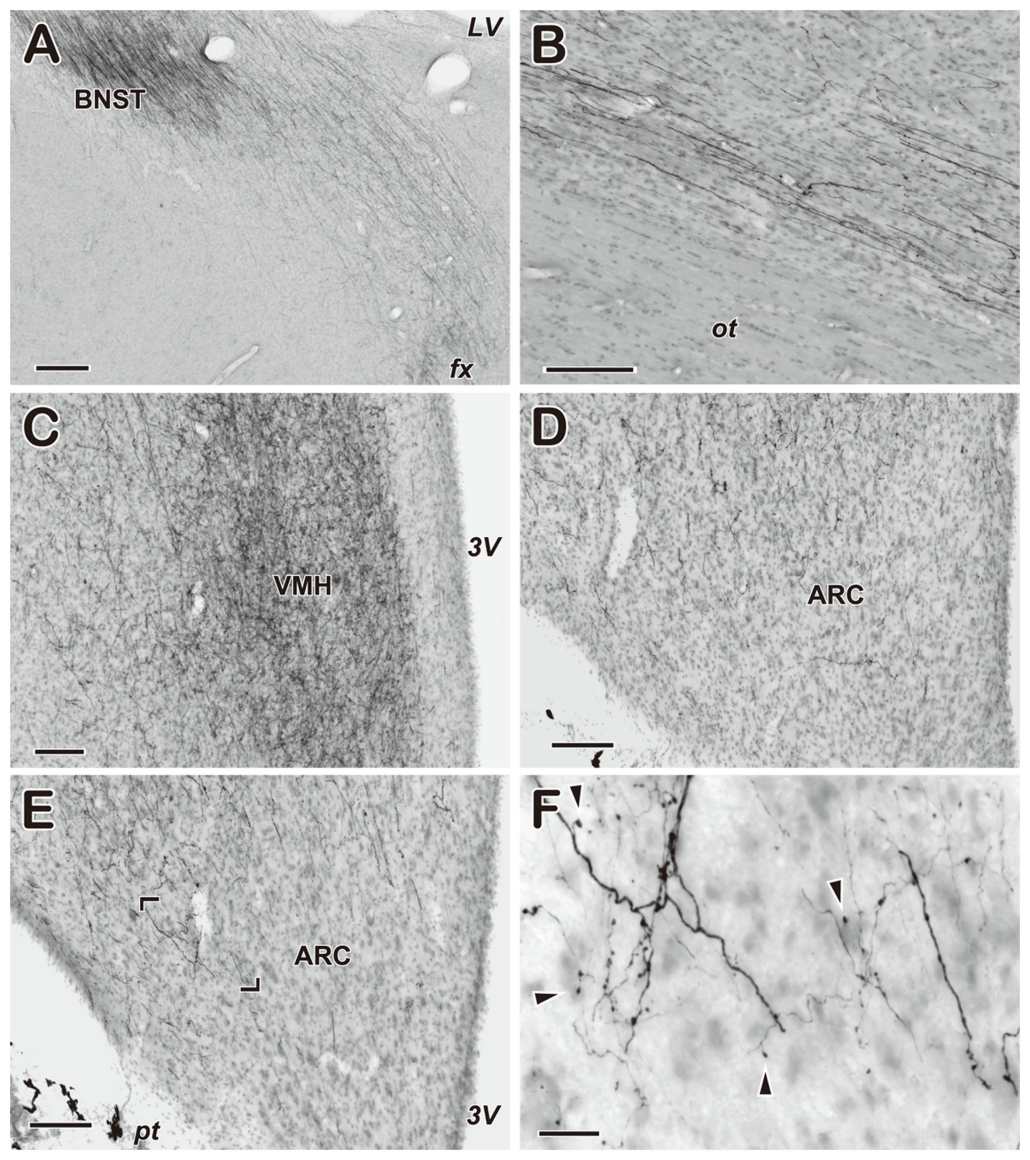
Photomicrographs of biotin dextran amine-labeled fibers. A, Labeled fibers forming a dense plexus in the bed nucleus of the stria terminalis. B, Labeled fibers coursing areas over the optic tract. C, Labeled fibers forming a dense plexus in the ventromedial nucleus of the hypothalamus. D, E, Labeled fibers distributed in the rostral (D) and middle region (E) of the arcuate nucleus. F, High magnification of the area indicated in E. Arrowheads indicate apparent terminal buttons. ARC, arcuate nucleus; BNST, bed nucleus of the stria terminalis; LHA; lateral hypothalamic area; VMH, ventromedial nucleus of the hypothalamus; *fx*, fornix; *LV*, lateral ventricle; *ot*, optic tract; *pt*, par tuberalis; *3V*, third ventricle. Scale bar: A = 300 µm; B-E =150 µm; F= 20 µm.

**Figure 4:**
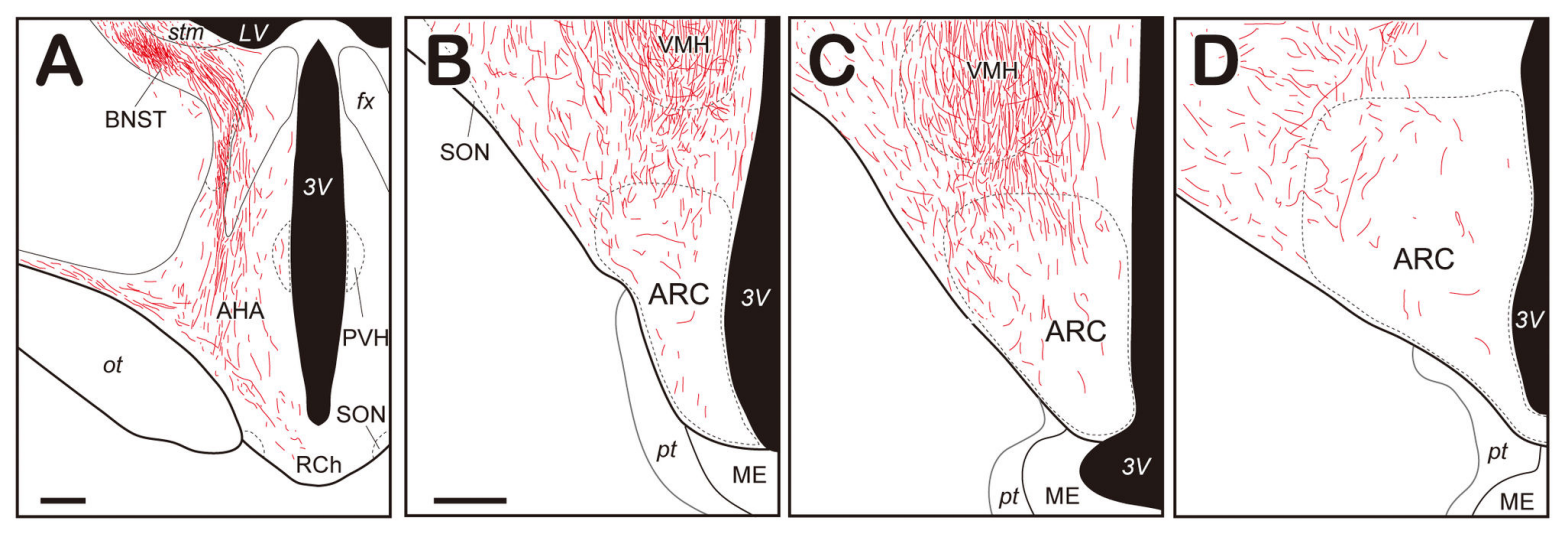
Schematic drawings of the distribution of labeled fibers. A, Frontal illustration of areas immediately rostral to the rostral edge of the arcuate nucleus (ARC). B–D, Frontal illustrations of the mediobasal hypothalamus containing the rostral (B), middle (C), and caudal (D) regions of the ARC. LHA; lateral hypothalamic area; ARC, arcuate nucleus; BNST, bed nucleus of the stria terminalis; PVH, paraventricular nucleus of the hypothalamus; SON, supraoptic nucleus; RCh, retrochiasmatic are; VMH, ventromedial nucleus of the hypothalamus; *fx*, fornix; *LV*, lateral ventricle; ME, median eminence; *ot*, optic tract; *pt*, par tuberalis; *stm*, stria medullaris of the thalamus; *3V*, third ventricle. Scale bar: A = 1 mm; B = 500 µm for B-D.

 Photomicrographs of BDA-labeled fibers in the hypothalamus and BNST are presented in Figure 3. The distribution of labeled fibers in a goat in which the injection was confined to the MeA (Figure 2B) is schematically shown in Figure 4. Efferent fibers of the MeA formed a dense plexus in the BNST and then coursed ventrally and medially to the hypothalamus (Figure 3A and 4A). Some efferent fibers of the MeA, perhaps not passing the BNST, coursed ventrally in areas over the optic tract (Figure 3B) and reached rostrally to areas ventral to the anterior hypothalamic area (Figure 4A) as well as caudally to the lateral hypothalamic area (LHA). They merged with descending labeled fibers from the BNST in the lateral region of the ARC. Labeled fibers were observed in several hypothalamic nuclei and were abundant in the ventromedial nucleus of the hypothalamus (VMH) and LHA. In particular, they formed a dense plexus in the VMH (Figure 3C). The ARC also contained a substantial number of labeled fibers (Figure 3D and 3E). Although labeled fibers were observed throughout the rostro-caudal extent of the nucleus, their density was markedly low in the caudal region (Figure 4B–D). Within the ARC, labeled fibers were predominantly distributed at its dorsal and lateral aspects. They were branched and possessed varicosities along with the axon and apparent terminal buttons (Figure 3F).

 The abovementioned distribution pattern of BDA-labeled fibers was similar among the goats that received BDA injection within the MeA (Figure 2). The density of labeled fibers in the ARC was relatively lower in the goat with the injection site located at the ventrolateral part of the MeA (Figure 2C) compared with that in the other 2 goats. Although some labeled fibers were occasionally observed in the contralateral side of the injection site, BDA-positive fibers were distributed predominantly in the ipsilateral side. As observed in previous studies using this tracer [49,50], retrogradely labeled cells were occasionally observed in all regions examined. In goats that received the BDA injection outside of the MeA (n = 3), labeled fibers were scarcely distributed in the hypothalamus, and few or no labeled fibers were observed in the ARC. 

**Figure 5:**
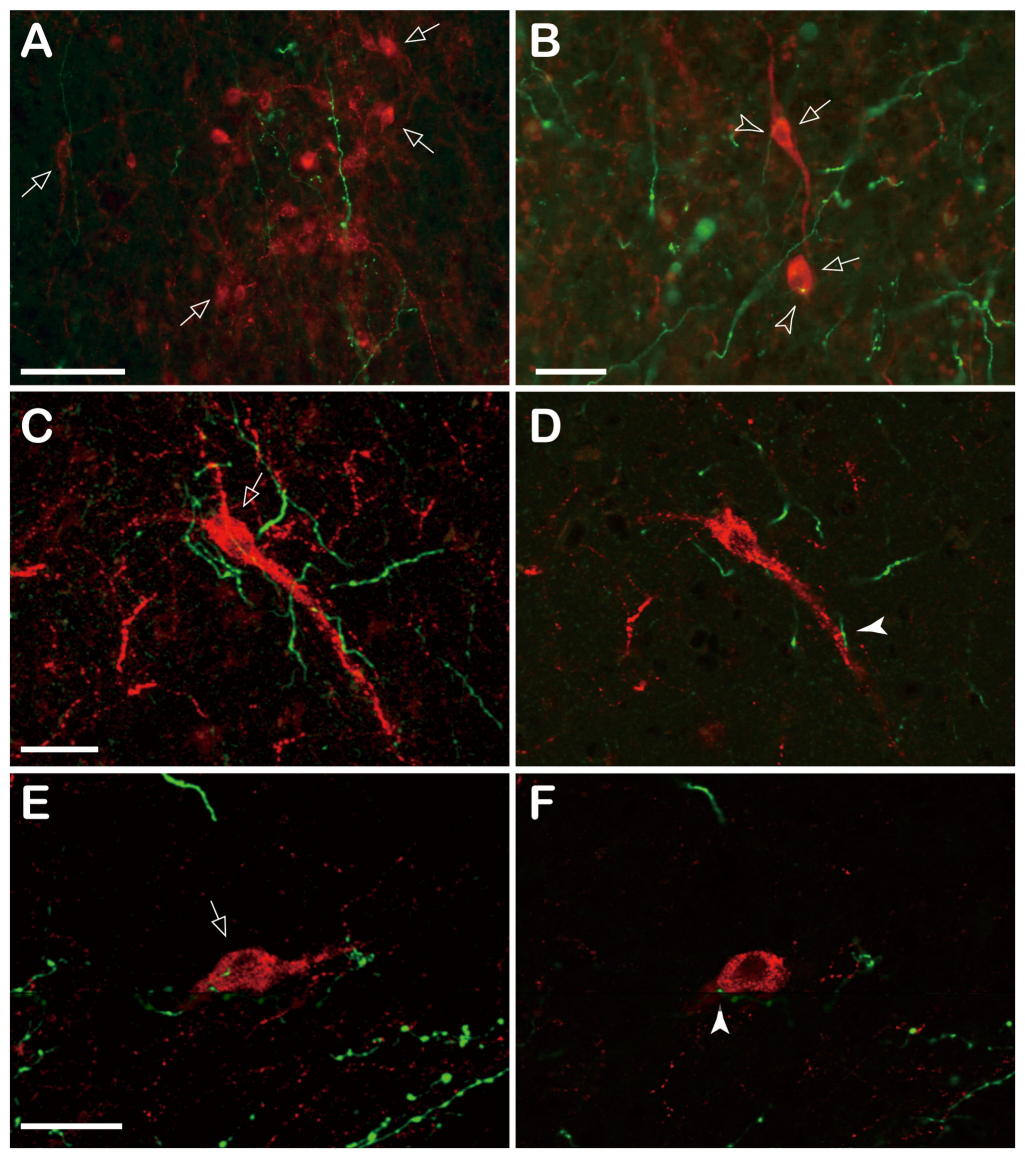
Photomicrographs of dual-labeling histochemical analyses for biotin dextran amine (BDA) and kisspeptin in the arcuate nucleus (ARC). A, B, Fluorescence microscopy images of BDA-labeled fibers (green) and kisspeptin-immunoreactive (-ir) neurons (red). C–F, Confocal microscopy images of BDA-labeled fibers (green) and kisspeptin-ir neurons (red). C, E, Stacked images of 15 serial confocal planes. D, F, A single 1-µm thick plane of pictures in C and E, respectively. Arrows indicate kisspeptin-ir neurons. Open arrowheads indicate BDA-labeled fibers running in close proximity to kisspeptin-ir neurons. Closed arrowheads indicate direct apposition of BDA-labeled fibers with kisspeptin-ir neurons. Scale bar: A = 100 µm; B = 50 µm; C = 40 µm for C and D; E = 40 µm for E and F.

 To examine whether ARC kisspeptin neurons are connected with the MeA, dual-labeling histochemical analyses for BDA and kisspeptin were performed. Fluorescence microscopic observations indicated that several BDA-positive fibers run and terminate in close vicinity with kisspeptin-immunoreactive (-ir) neurons in the ARC (Figure 5A). It appeared that some appose kisspeptin-ir neurons (Figure 5B). Such occurrences were observed approximately 0–3 instances per slide. However, based on fluorescence microscopic observation, it was unclear whether they represented direct contact between BDA-positive fibers and kisspeptin-ir neurons. Therefore, several slides were analyzed further using confocal microscopy. These analyses revealed that some BDA fibers actually appose kisspeptin-ir neural processes (perhaps dendrites) (Figure 5C and 5D) or kisspeptin-ir cell bodies (Figure 5E and 5F). The occurrence of apposition was, however, relatively low.

## Discussion

 Although it has long been accepted that the central target of the male pheromone signal is the putative hypothalamic GnRH pulse generator [2,4,10,11], the precise neural mechanisms underlying pheromone action remained unclear. In the present study, we demonstrated in goats that pheromone action on GnRH pulse generator activity depends on entirely NKB signaling, and ARC kisspeptin neurons receive projections from the MeA. NKB signaling is suggested to play a pivotal role in the GnRH pulse generation by ARC kisspeptin neurons [24,38,39,46,51]. Moreover, the MeA is considered to be a key neural substrate that relays pheromone signals to target nuclei in the hypothalamus [41-43,52]. Considering these findings, the present results strongly suggest that the male pheromone signal is transmitted to, and processed in, a subset of ARC kisspeptin/NKB neurons, triggering pulsatile GnRH/LH secretion. 

 Consistent with previous studies [10,19-22], an MUA volley was induced within 30 s after exposure to the pheromone in all vehicle-treated control goats in this study. However, when goats were treated with the NK3R antagonist SB222200, neither the MUA volley nor the LH pulse was observed upon pheromone exposure (Figure 1). These results demonstrate that NK3R antagonists negate the action of the male pheromone on the GnRH pulse generator, thereby suppressing pulsatile LH secretion in goats. Although NK3Rs are distributed in the central as well as the peripheral nervous system [53], the action of peripherally administered SB222200 on the male effect might be exerted centrally as it affected MUA volley induction. There are 2 possible sites of action for the antagonist in the brain. One is the ARC and the other is the retrochiasmatic area (RCh). It has been demonstrated in sheep that a subset of neurons in the RCh contain NK3R [54], and local administration of an NK3R agonist into the RCh dramatically increases LH secretion [55]. Therefore, NK3R-containing RCh neurons may be involved in the control of GnRH secretion and, thus, in the stimulatory action of the pheromone on GnRH/LH release. However, this seems unlikely because activation of NKB signaling in the RCh results in a surge-like LH release [55], whereas the pheromone induces pulsatile small amplitude LH secretion. It is more likely that SB222200 negates pheromone action by blocking NKB signaling in ARC kisspeptin neurons. A majority of ARC kisspeptin/NKB neurons co-expresses NK3R in sheep [54] and goats [56]. The observation that central administration of NKB activated approximately 70% of ARC kisspeptin/NKB neurons and induced pulse-like LH secretion in seasonally anestrous ewes [30] suggests that NKB signaling in ARC kisspeptin/NKB neurons is associated with pulsatile GnRH/LH secretion. Moreover, the electrode monitoring MUA was targeted to ARC kisspeptin/NKB neurons in the present and previous [10] studies, where it was unambiguously demonstrated that the pheromone induces the MUA volley. 

 In the present study, the intervolley interval after SB222200 administration was significantly prolonged as compared with that in the control period. This result indicates that the antagonist suppressed the pheromone-induced as well as the spontaneously occurring MUA volleys. Therefore, the effect of the antagonist might not be specific for pheromone action, rather may be an overall suppression of GnRH pulse generator activity itself. Recent studies in rats [51] and goats [46] have demonstrated that ARC kisspeptin/NKB neurons are interconnected and suggest that NKB signaling may play a role in synchronized bursting activities in such a neural network. In this context, it can be assumed that the antagonist prevented synchronized bursting among ARC kisspeptin/NKB neurons, thereby negating pheromone action and suppressing the occurrence of spontaneous MUA volleys. A recent study showed that infusion of a kisspeptin receptor antagonist [31] into the lateral ventricle completely blocked the effect of ram exposure on LH secretion in seasonally anestrous ewes [57]. Therefore, it is likely that kisspeptin signaling, as well as NKB signaling, plays critical roles in the central mechanism of the male effect, in which the pheromone signal is translated to pulsatile GnRH/LH secretion. 

 The present anterograde tracer study demonstrated that the MeA sends projections to several hypothalamic nuclei, including the ARC (Figure 4). The pathway and distribution of the efferent fibers of the MeA observed in goats are comparable to those demonstrated in rats [58,59] and hamsters [60], suggesting that these neural pathways are highly conserved among mammals. Efferent fibers of the MeA were branched and possessed varicosities and terminal buttons in the ARC (Figure 3F), indicating that the ARC is the terminal field for MeA efferent fibers. Moreover, dual-labeling histochemical analyses revealed that kisspeptin neurons in the ARC received apposition of fibers originating from the MeA (Figure 5D and 5F). These results indicate that the MeA sends projections to ARC kisspeptin/NKB neurons.

 Although the results of the tracer study clearly demonstrate the presence of a neural connection between the MeA and ARC kisspeptin/NKB neurons, the anatomical data do not provide direct insights regarding whether such a neural pathway is indeed involved in male pheromone signal transduction. However, several lines of evidence suggest that this may be the case. First, all putative pheromone receptors, V1Rs, identified thus far in goats are expressed in both vomeronasal and olfactory sensory neurons [61,62], and the MeA receives inputs from these 2 types of sensory neurons via the accessory and main olfactory bulbs, respectively [63,64]. Second, the MeA is proposed to be a key nucleus that relays pheromone signals to target nuclei in the hypothalamus [41-43]. Third, exposure of seasonally anestrous ewes to rams results in the induction of c-Fos, a marker of neuronal activation, in MeA neurons [65] as well as in ARC kisspeptin/NKB neurons [57]. Finally, the male effect in goats and sheep depends on NKB (this study) and kisspeptin [57] signaling, both of which are associated with ARC kisspeptin/NKB neurons. Therefore, it is possible that the MeA relays the male pheromone signal to a subset of ARC kisspeptin/NKB neurons, where it is translated to neural activity that elicits pulsatile GnRH release through NKB and kisspeptin signaling.

 Although a substantial number of MeA efferent fibers terminate in the ARC, the direct apposition of these fibers on kisspeptin cell bodies was observed in a few instances. This raises the possibility that other neurons in the ARC could receive the pheromone signal from the MeA and relay the signal to ARC kisspeptin/NKB neurons. ARC kisspeptin neurons are known to receive inputs from neuropeptide Y (NPY), proopiomelanocortin, and dopamine neurons in sheep [66,67]. The cell bodies of these neurotransmitters are also known to reside in the ARC. Therefore, the pheromone action may be mediated by either of these neurotransmitters. However, this is unlikely because it has been shown that NPY suppresses GnRH pulse generator activity in goats [18], and dopamine acts to inhibit LH secretion in anestrous ewes [67]. Moreover, although there is evidence that central administration of a melanocortin receptor agonist slightly stimulates GnRH pulse generator activity, this effect was observed 10–15 min after administration [68], which is in contrast to the rapid action of the pheromone (within 1 min). Thus, whether other neurons, in addition to kisspeptin neurons, are involved in pheromone action remains unclear. Alternatively, pheromone induction of the MUA volley and LH pulse may be mediated by a small subset of ARC kisspeptin neurons. ARC kisspeptin/NKB neurons are interconnected with each other to form a neuronal circuit [46,51]. Studies have suggested that the initial activation aroused in a subset of kisspeptin neurons would be amplified and propagated among cells within this circuit to evoke synchronized bursting of kisspeptin neurons [24,34,38]. In this case, ARC kisspeptin/NKB neurons may not necessarily receive rich projections from the MeA, and this may explain why apposition of the MeA/BNST projections on kisspeptin neurons was observed only in a few instances.

 In conclusion, we demonstrated in the present study that the MUA volley and LH responses to male pheromone are thoroughly suppressed by administration of an NKB receptor antagonist. We also demonstrated that a subset of ARC kisspeptin/NKB neurons receive efferent projections from the MeA in goats. These results suggest that the chemosensory signal of the male pheromone is conveyed via the MeA to ARC kisspeptin/NKB neurons, where the signal stimulates pulse generator activity through an NKB signaling-mediated mechanism. 

## Supporting Information

Figure S1 **Multiple-unit activity (MUA) profiles and intervolley intervals during the 4-h experimental period (2-h control period plus 2-h blood sampling period) in three representative ovariectomized goats.** Goats were exposed to the male pheromone in the absence or presence of the NK3R antagonist SB222200. The timing of pheromone exposure is indicated by an arrow. Vehicle (open arrowheads) or SB222200 (closed arrowheads) were injected intravenously twice (at the first and fourth blood sampling points) after the preceding MUA volley. Small parts of MUA data in the vehicle treatment of a goat #612 are missing due to a technical problem.
(TIF)

## References

[1] Schinckel PG (1954) The effect of the presence of the ram on the ovarian activity of the ewe. Aust J Agric Res 5: 465-469. doi:10.1071/AR9540465.

[2] Martin GB, Oldham CM, Cognie Y, Pearce DT (1986) The physiological-responses of anovulatory ewes to the introduction of rams - a review. Livest Prod Sci 15: 219-247. doi:10.1016/0301-6226(86)90031-X.

[3] Chemineau P (1987) Possibilities for using bucks to stimulate ovarian and estrous cycles in anovulatory goats - a review. Livest Prod Sci 17: 135-147. doi:10.1016/0301-6226(87)90059-5.

[4] Delgadillo JA, Gelez H, Ungerfeld R, Hawken PAR, Martin GB (2009) The 'male effect' in sheep and goats-Revisiting the dogmas. Behav Brain Res 200: 304-314. doi:10.1016/j.bbr.2009.02.004. PubMed: 19374015. 19374015

[5] Gelez H, Fabre-Nys C (2004) The "male effect" in sheep and goats: a review of the respective roles of the two olfactory systems. Horm Behav 46: 257-271. doi:10.1016/j.yhbeh.2004.05.002. PubMed: 15325227. 15325227

[6] Signoret JP (1982) The male effect. Recherche 13: 512-514.

[7] Walkdenbrown SW, Restall BJ, Henniawati (1993) The male effect in the Australian Cashmere goat. 1. Ovarian and behavioral response of seasonally anovulatory does following the introduction of bucks. Anim Reprod Sci 32: 41-53. doi:10.1016/0378-4320(93)90056-W.

[8] Knight TW, Lynch PR (1980) Source of ram pheromones that stimulate ovulation in the ewe. Anim Reprod Sci 3: 133-136. doi:10.1016/0378-4320(80)90040-8.

[9] Claus R, Over R, Dehnhard M (1990) Effect of male odour on LH secretion and the induction of ovulation in seasonally anoestrous goats. Anim Reprod Sci 22: 27-38. doi:10.1016/0378-4320(90)90035-E.

[10] Murata K, Wakabayashi Y, Sakamoto K, Tanaka T, Takeuchi Y et al. (2011) Effects of brief exposure of male pheromone on multiple-unit activity at close proximity to kisspeptin neurons in the goat arcuate nucleus. J Reprod Dev 57: 197-202. doi:10.1262/jrd.10-070E. PubMed: 21123964. 21123964

[11] Okamura H, Murata K, Sakamoto K, Wakabayashi Y, Ohkura S et al. (2010) Male effect pheromone tickles the gonadotrophin-releasing hormone pulse generator. J Neuroendocrinol 22: 825-832. doi:10.1111/j.1365-2826.2010.02037.x. PubMed: 20646176. 20646176

[12] Karsch FJ (1984) The hypothalamus and anterior pituitary gland. In: Austin CR Short RV Reproduction in mammals, Vol.3, Hormonal Control of Reproduction, 2nd ed. Cambridge: Cambridge University Press pp. 1-20.

[13] Knobil E (1981) Patterns of hypophysiotropic signals and gonadotropin-secretion in the rhesus monkey. Biol Reprod 24: 44-49. doi:10.1095/biolreprod24.1.44. PubMed: 6781549. 6781549

[14] O’Byrne KT, Chen MD, Nishihara M, Williams CL, Thalabard JC et al. (1993) Ovarian control of gonadotropin hormone-releasing hormone pulse generator activity in the rhesus monkey: duration of the associated hypothalamic signal. Neuroendocrinology 57: 588-592. doi:10.1159/000126411. PubMed: 8367027. 8367027

[15] Nishihara M, Hiruma H, Kimura F (1991) Interactions between the noradrenergic and opioid peptidergic systems in controlling the electrical activity of luteinizing hormone-releasing hormone pulse generator in ovariectomized rats. Neuroendocrinology 54: 321-326. doi:10.1159/000125909. PubMed: 1758574. 1758574

[16] Kinsey-Jones JS, Li XF, Luckman SM, O'Byrne KT (2008) Effects of kisspeptin-10 on the electrophysiological manifestation of gonadotropin-releasing hormone pulse generator activity in the female rat. Endocrinology 149: 1004-1008. PubMed: 18063679. 18063679 10.1210/en.2007-1505

[17] Mori Y, Nishihara M, Tanaka T, Shimizu T, Yamaguchi M et al. (1991) Chronic recording of electrophysiological manifestation of the hypothalamic gonadotropin-releasing hormone pulse generator activity in the goat. Neuroendocrinology 53: 392-395. doi:10.1159/000125746. PubMed: 2046871. 2046871

[18] Ichimaru T, Mori Y, Okamura H (2001) A possible role of neuropeptide Y as a mediator of undernutrition to the hypothalamic gonadotropin-releasing hormone pulse generator in goats. Endocrinology 142: 2489-2498. doi:10.1210/en.142.6.2489. PubMed: 11356698. 11356698

[19] Hamada T, Nakajima M, Takeuchi Y, Mori Y (1996) Pheromone-induced stimulation of hypothalamic gonadotropin-releasing hormone pulse generator in ovariectomized, estrogen-primed goats. Neuroendocrinology 64: 313-319. doi:10.1159/000127134. PubMed: 8895861. 8895861

[20] Iwata E, Wakabayashi Y, Kakuma Y, Kikusui T, Takeuchi Y et al. (2000) Testosterone-dependent primer pheromone production in the sebaceous gland of male goat. Biol Reprod 62: 806-810. doi:10.1095/biolreprod62.3.806. PubMed: 10684827. 10684827

[21] Kakuma Y, Ichimaru T, Yonezawa T, Momozawa Y, Hashizume C et al. (2007) Androgen induces production of male effect pheromone in female goats. J Reprod Dev 53: 829-834. doi:10.1262/jrd.19013. PubMed: 17460391. 17460391

[22] Ichimaru T, Mogi K, Ohkura S, Mori Y, Okamura H (2008) Exposure to ram wool stimulates gonadotropin-releasing hormone pulse generator activity in the female goat. Anim Reprod Sci 106: 361-368. doi:10.1016/j.anireprosci.2007.05.012. PubMed: 17573212. 17573212

[23] Kano Y, Sawasaki T, Oyama T (1977) Biological characteristics of miniature "Shiba" goats. Exp Anim 26: 239-246. 908359

[24] Wakabayashi Y, Nakada T, Murata K, Ohkura S, Mogi K et al. (2010) Neurokinin B and dynorphin A in kisspeptin neurons of the arcuate nucleus participate in generation of periodic oscillation of neural activity driving pulsatile gonadotropin-releasing hormone secretion in the goat. J Neurosci 30: 3124–3132. doi:10.1523/JNEUROSCI.5848-09.2010. PubMed: 20181609. 20181609 PMC6633939

[25] de Roux N, Genin E, Carel JC, Matsuda F, Chaussain JL et al. (2003) Hypogonadotropic hypogonadism due to loss of function of the KiSS1-derived peptide receptor GPR54. Proc Natl Acad Sci U S A 100: 10972-10976. doi:10.1073/pnas.1834399100. PubMed: 12944565. 12944565 PMC196911

[26] Seminara SB, Messager S, Chatzidaki EE, Thresher RR, Acierno JS Jr. et al. (2003) The GPR54 gene as a regulator of puberty. N Engl J Med 349: 1614-1627. doi:10.1056/NEJMoa035322. PubMed: 14573733. 14573733

[27] Topaloglu AK, Reimann F, Guclu M, Yalin AS, Kotan LD et al. (2009) TAC3 and TACR3 mutations in familial hypogonadotropic hypogonadism reveal a key role for Neurokinin B in the central control of reproduction. Nat Genet 41: 354-358. doi:10.1038/ng.306. PubMed: 19079066. 19079066 PMC4312696

[28] Oakley AE, Clifton DK, Steiner RA (2009) Kisspeptin signaling in the brain. Endocr Rev 30: 713-743. doi:10.1210/er.2009-0005. PubMed: 19770291. 19770291 PMC2761114

[29] Ramaswamy S, Seminara SB, Ali B, Ciofi P, Amin NA et al. (2010) Neurokinin B stimulates GnRH release in the male monkey (Macaca mulatta) and is colocalized with kisspeptin in the arcuate nucleus. Endocrinology 151: 4494-4503. doi:10.1210/en.2010-0223. PubMed: 20573725. 20573725 PMC2940495

[30] Sakamoto K, Murata K, Wakabayashi Y, Yayou K, Ohkura S et al. (2012) Central Administration of Neurokinin B Activates Kisspeptin/NKB Neurons in the Arcuate Nucleus and Stimulates Luteinizing Hormone Secretion in Ewes During the Non-Breeding Season. J Reprod Dev 58: 700-706. doi:10.1262/jrd.2011-038. PubMed: 22972185. 22972185

[31] Roseweir AK, Kauffman AS, Smith JT, Guerriero KA, Morgan K et al. (2009) Discovery of potent kisspeptin antagonists delineate physiological mechanisms of gonadotropin regulation. J Neurosci 29: 3920-3929. doi:10.1523/JNEUROSCI.5740-08.2009. PubMed: 19321788. 19321788 PMC3035813

[32] Smith JT, Li Q, Yap KS, Shahab M, Roseweir AK et al. (2011) Kisspeptin is essential for the full preovulatory LH surge and stimulates GnRH release from the isolated ovine median eminence. Endocrinology 152: 1001-1012. doi:10.1210/en.2010-1225. PubMed: 21239443. 21239443

[33] Noritake K, Matsuoka T, Ohsawa T, Shimomura K, Sanbuissho A et al. (2011) Involvement of neurokinin receptors in the control of pulsatile luteinizing hormone secretion in rats. J Reprod Dev 57: 409-415. doi:10.1262/jrd.11-002S. PubMed: 21358144. 21358144

[34] Navarro VM, Gottsch ML, Chavkin C, Okamura H, Clifton DK et al. (2009) Regulation of gonadotropin-releasing hormone secretion by kisspeptin/dynorphin/neurokinin B neurons in the arcuate nucleus of the mouse. J Neurosci 29: 11859-11866. doi:10.1523/JNEUROSCI.1569-09.2009. PubMed: 19776272. 19776272 PMC2793332

[35] Navarro VM, Castellano JM, McConkey SM, Pineda R, Ruiz-Pino F et al. (2011) Interactions between kisspeptin and neurokinin B in the control of GnRH secretion in the female rat. Am J Physiol Endocrinol Metab 300: E202-E210. doi:10.1152/ajpendo.00517.2010. PubMed: 21045176. 21045176 PMC3774070

[36] Goodman RL, Lehman MN, Smith JT, Coolen LM, De Oliveira CVR et al. (2007) Kisspeptin neurons in the arcuate nucleus of the ewe express both dynorphin a and neurokinin B. Endocrinology 148: 5752-5760. doi:10.1210/en.2007-0961. PubMed: 17823266. 17823266

[37] Lehman MN, Coolen LM, Goodman RL (2010) Minireview: kisspeptin/neurokinin B/dynorphin (KNDy) cells of the arcuate nucleus: a central node in the control of gonadotropin-releasing hormone secretion. Endocrinology 151: 3479-3489. doi:10.1210/en.2010-0022. PubMed: 20501670. 20501670 PMC2940527

[38] Maeda K, Ohkura S, Uenoyama Y, Wakabayashi Y, Oka Y et al. (2010) Neurobiological mechanisms underlying GnRH pulse generation by the hypothalamus. Brain Res 1364: 103-115. doi:10.1016/j.brainres.2010.10.026. PubMed: 20951683. 20951683

[39] Rance NE, Krajewski SJ, Smith MA, Cholanian M, Dacks PA (2010) Neurokinin B and the hypothalamic regulation of reproduction. Brain Res 1364: 116-128. doi:10.1016/j.brainres.2010.08.059. PubMed: 20800582. 20800582 PMC2992576

[40] Ohkura S, Takase K, Matsuyama S, Mogi K, Ichimaru T et al. (2009) Gonadotrophin-releasing hormone pulse generator activity in the hypothalamus of the goat. J Neuroendocrinol 21: 813-821. doi:10.1111/j.1365-2826.2009.01909.x. PubMed: 19678868. 19678868

[41] Brennan PA (2001) The vomeronasal system. Cell Mol Life Sci 58: 546-555. doi:10.1007/PL00000880. PubMed: 11361090. 11361090 PMC11146473

[42] Keverne EB (1983) Pheromonal influences on the endocrine regulation of reproduction. Trends Neurosci 6: 381-384. doi:10.1016/0166-2236(83)90170-4.

[43] Li CS, Kaba H, Saito H, Seto K (1990) Neural mechanisms underlying the action of primer pheromones in mice. Neuroscience 36: 773-778. doi:10.1016/0306-4522(90)90019-Z. PubMed: 2234409. 2234409

[44] Beltramino C, Taleisnik S (1978) Facilitatory and inhibitory effects of electrochemical stimulation of the amygdala on the release of luteinizing hormone. Brain Res 144: 95-107. doi:10.1016/0006-8993(78)90437-7. PubMed: 565243. 565243

[45] Suganuma C, Kuroiwa T, Tanaka T, Kamomae H (2007) Changes in the ovarian dynamics and endocrine profiles in goats treated with a progesterone antagonist during the early luteal phase of the estrous cycle. Anim Reprod Sci 101: 285-294. doi:10.1016/j.anireprosci.2006.09.015. PubMed: 17027203. 17027203

[46] Wakabayashi Y, Yamamura T, Sakamoto K, Mori Y, Okamura H (2012) Electrophysiological and morphological evidence for synchronized GnRH pulse generator activity among kisspeptin/neurokinin B/dynorphin A (KNDy) neurons in goats. J Reprod Dev 59: 40-48. PubMed: 23080371. 23080371 10.1262/jrd.2012-136 PMC3943231

[47] Watson RE Jr., Wiegand SJ, Clough RW, Hoffman GE (1986) Use of cryoprotectant to maintain long-term peptide immunoreactivity and tissue morphology. Peptides 7: 155-159. doi:10.1016/0196-9781(86)90179-8. PubMed: 3520509. 3520509

[48] Zuccolilli GO, Hayashi S, Mori Y (1995) Hypothalamic structures of the goat on stereotaxic coordinates. J Vet Med Sci 57: 459-467. doi:10.1292/jvms.57.459. PubMed: 7548398. 7548398

[49] Pompolo S, Rawson JA, Clarke IJ (2001) Projections from the arcuate/ventromedial region of the hypothalamus to the preoptic area and bed nucleus of stria terminalis in the brain of the ewe; lack of direct input to gonadotropin-releasing hormone neurons. Brain Res 904: 1-12. doi:10.1016/S0006-8993(01)02372-1. PubMed: 11516406. 11516406

[50] Pompolo S, Ischenko O, Pereira A, Iqbal J, Clarke IJ (2005) Evidence that projections from the bed nucleus of the stria terminalis and from the lateral and medial regions of the preoptic area provide input to gonadotropin releasing hormone (GNRH) neurons in the female sheep brain. Neuroscience 132: 421-436. doi:10.1016/j.neuroscience.2004.12.042. PubMed: 15802194. 15802194

[51] Krajewski SJ, Burke MC, Anderson MJ, McMullen NT, Rance NE (2010) Forebrain projections of arcuate neurokinin B neurons demonstrated by anterograde tract-tracing and monosodium glutamate lesions in the rat. Neuroscience 166: 680-697. doi:10.1016/j.neuroscience.2009.12.053. PubMed: 20038444. 20038444 PMC2823949

[52] Lehman MN, Winans SS, Powers JB (1980) Medial nucleus of the amygdala mediates chemosensory control of male hamster sexual behavior. Science 210: 557-560. doi:10.1126/science.7423209. PubMed: 7423209. 7423209

[53] Almeida TA, Rojo J, Nieto PM, Pinto FM, Hernandez M et al. (2004) Tachykinins and tachykinin receptors: Structure and activity relationships. Curr Med Chem 11: 2045-2081. doi:10.2174/0929867043364748. PubMed: 15279567. 15279567

[54] Amstalden M, Coolen LM, Hemmerle AM, Billings HJ, Connors JM et al. (2010) Neurokinin 3 receptor immunoreactivity in the septal region, preoptic area and hypothalamus of the female sheep: Colocalisation in neurokinin B cells of the arcuate nucleus but not in gonadotrophin-releasing hormone neurones. J Neuroendocrinol 22: 1-12. doi:10.1111/j.1365-2826.2009.01930.x. PubMed: 19912479. 19912479 PMC2821793

[55] Billings HJ, Connors JM, Altman SN, Hileman SM, Holaskova I et al. (2010) Neurokinin B acts via the neurokinin-3 receptor in the retrochiasmatic area to stimulate luteinizing hormone secretion in sheep. Endocrinology 151: 3836-3846. doi:10.1210/en.2010-0174. PubMed: 20519368. 20519368 PMC2940514

[56] Wakabayashi Y, Yamamura T, Ohkura S, Homma T, Sakamoto K et al Senktide, a neurokinin B receptor agonist, stimulates pulsatile LH secretion through a mechanism mediated by the GnRH pulse generator in goats. In: The 42nd annual meeting of the Society for Neuroscience, New Orleans, USA, October 13-17.

[57] De Bond JA, Li Q, Millar RP, Clarke IJ, Smith JT (2013) Kisspeptin signaling is required for the luteinizing hormone response in anestrous ewes following the introduction of males. PLOS ONE 8: e57972. doi:10.1371/journal.pone.0057972. PubMed: 23469121. 23469121 PMC3585258

[58] Canteras NS, Simerly RB, Swanson LW (1995) Organization of projections from the medial nucleus of the amygdala: a PHAL study in the rat. J Comp Neurol 360: 213-245. doi:10.1002/cne.903600203. PubMed: 8522644. 8522644

[59] Dong HW, Swanson LW (2006) Projections from bed nuclei of the stria terminalis, dorsomedial nucleus: implications for cerebral hemisphere integration of neuroendocrine, autonomic, and drinking responses. J Comp Neurol 494: 75-107. doi:10.1002/cne.20790. PubMed: 16304681. 16304681 PMC2707828

[60] Gomez DM, Newman SW (1992) Differential projections of the anterior and posterior regions of the medial amygdaloid nucleus in the Syrian hamster. J Comp Neurol 317: 195-218. doi:10.1002/cne.903170208. PubMed: 1573064. 1573064

[61] Wakabayashi Y, Mori Y, Ichikawa M, Yazaki K, Hagino-Yamagishi K (2002) A putative pheromone receptor gene is expressed in two distinct olfactory organs in goats. Chem Sens 27: 207-213. doi:10.1093/chemse/27.3.207. 11923183

[62] Ohara H, Nikaido M, Date-Ito A, Mogi K, Okamura H et al. (2009) Conserved repertoire of orthologous vomeronasal type 1 receptor genes in ruminant species. BMC Evol Biol 9: 233. doi:10.1186/1471-2148-9-233. PubMed: 19751533. 19751533 PMC2758851

[63] Scalia F, Winans SS (1975) The differential projections of the olfactory bulb and accessory olfactory bulb in mammals. J Comp Neurol 161: 31-55. doi:10.1002/cne.901610105. PubMed: 1133226. 1133226

[64] Lehman MN, Winans SS (1982) Vomeronasal and olfactory pathways to the amygdala controlling male hamster sexual behavior: autoradiographic and behavioral analyses. Brain Res 240: 27-41. doi:10.1016/0006-8993(82)90641-2. PubMed: 7093718. 7093718

[65] Gelez H, Fabre-Nys C (2006) Neural pathways involved in the endocrine response of anestrous ewes to the male or its odor. Neuroscience 140: 791-800. doi:10.1016/j.neuroscience.2006.02.066. PubMed: 16650943. 16650943

[66] Backholer K, Smith JT, Rao A, Pereira A, Iqbal J et al. (2010) Kisspeptin cells in the ewe brain respond to leptin and communicate with neuropeptide Y and proopiomelanocortin cells. Endocrinology 151: 2233-2243. doi:10.1210/en.2009-1190. PubMed: 20207832. 20207832

[67] Goodman RL, Maltby MJ, Millar RP, Hileman SM, Nestor CC et al. (2012) Evidence that dopamine acts via kisspeptin to hold GnRH pulse frequency in check in anestrous ewes. Endocrinology 153: 5918-5927. doi:10.1210/en.2012-1611. PubMed: 23038740. 23038740 PMC3512065

[68] Matsuyama S, Ohkura S, Sakurai K, Tsukamura H, Maeda K et al. (2005) Activation of melanocortin receptors accelerates the gonadotropin-releasing hormone pulse generator activity in goats. Neurosci Lett 383: 289-294. doi:10.1016/j.neulet.2005.04.026. PubMed: 15955423. 15955423

