# Supplementary figures and images for: A Population of Kisspeptin/Neurokinin B Neurons in the Arcuate Nucleus May Be the Central Target of the Male Effect Phenomenon in Goats
Source: PLoS One. 2013 Nov 18;8(11):e81017. doi: 10.1371/journal.pone.0081017 (PMC3832416; doi:10.1371/journal.pone.0081017)

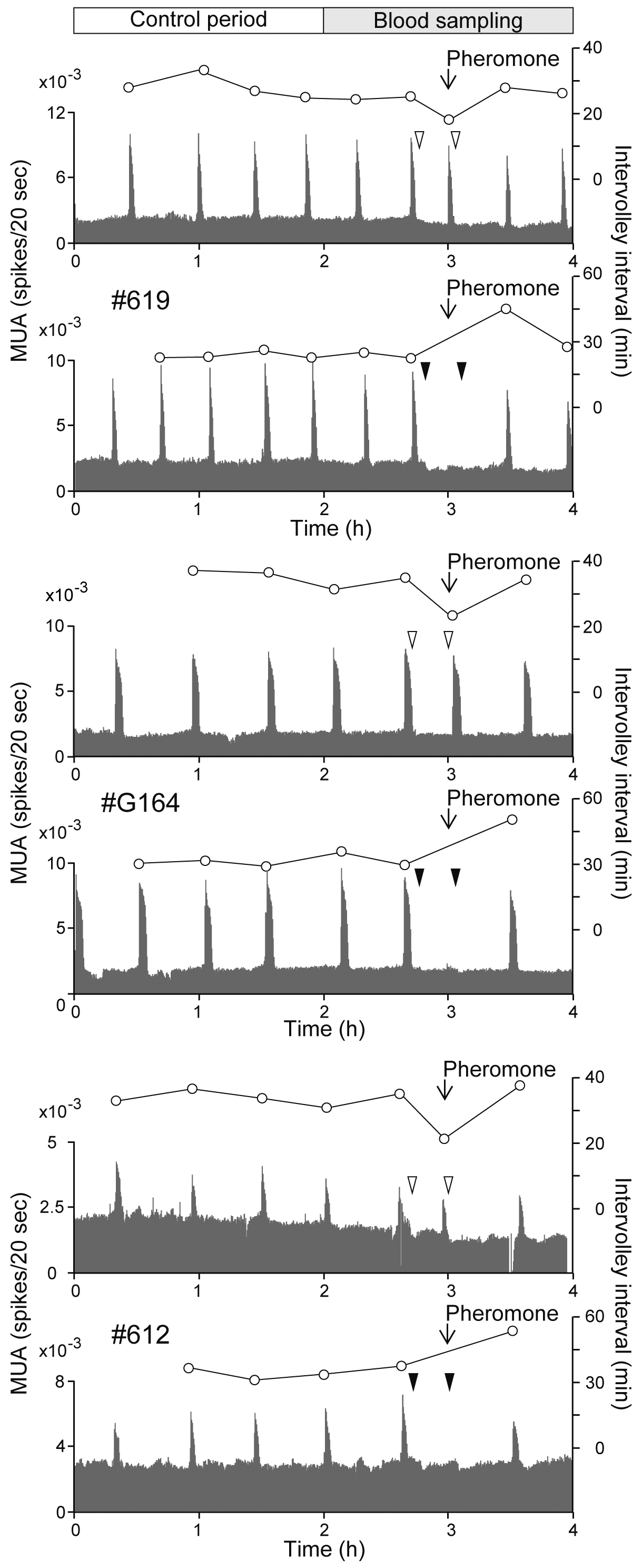

Supplement: Figure S1 — Multiple-unit activity (MUA) profiles and intervolley intervals during the 4-h experimental period (2-h control period plus 2-h blood sampling period) in three representative ovariectomized goats. Goats were exposed to the male pheromone in the absence or presence of the NK3R antagonist SB222200. The timing of pheromone exposure is indicated by an arrow. Vehicle (open arrowheads) or SB222200 (closed arrowheads) were injected intravenously twice (at the first and fourth blood sampling points) after the preceding MUA volley. Small parts of MUA data in the vehicle treatment of a goat #612 are missing due to a technical problem. (TIF) [file pone.0081017.s001.tif]
